# ImageJ Analysis for Transabdominal Endometrial Sonography of Single Saudi Females: A Cohort Study

**DOI:** 10.2174/0115734056434787251008053407

**Published:** 2025-10-14

**Authors:** Mahasin G. Hassan, Huda Alnafa, Nourah Alsofyan, Reham Alsulimi, Sumayah Alsllal, Noura Abaalkhail, Mona Alzurayr, Hessah Alamr, Tasneem SA Elmahdi, Asma Aldahes, Mayson Wanasi, Halima Hawesa

**Affiliations:** 1 Department of Radiological Sciences, College of Health and Rehabilitation Sciences, Princess Nourah bint Abdulrahman University, Riyadh, Saudi Arabia; 2 Department of Diagnostic Radiologic Technology, Faculty of Applied Medical Sciences, Taibah University, Al-Madinah Al-Munawara, Saudi Arabia; 3 Department of Radiological Sciences, Al-Ghad International Colleges for Applied Sciences, Madina, Saudi Arabia

**Keywords:** Transabdominal sonography, ImageJ analysis, Endometrium, Histogram, Single saudi females, Steroid hormones

## Abstract

**Background:**

Gynecological assessment of single females in some countries, where transvaginal ultrasound can not be performed, presents a challenge. This study proposed using computer-assisted analysis (ImageJ software) to assess its feasibility for endometrial analysis and consequently enhance the diagnostic value of transabdominal ultrasound.

**Materials and Methods:**

This pilot normative cohort study was conducted among 20 single healthy volunteers recruited at Princess Nourah University (PNU) ultrasound lab from November 2022 to April 2023. Participants were followed throughout their entire menstrual cycle and underwent a transabdominal ultrasound in the 4 menstrual phases. Sonographs were analyzed using the ImageJ program, and the data were analyzed with SPSS software.

**Results:**

The mean age of the participants was 21 years (± 0.9), and the average menstrual cycle length was 29.65 days (±2.18). The endometrium measured 0.33 cm (±0.137), 0.63 cm (±0.172), 0.89 cm (±0.167), and 1.06 cm (±0.19) in the menstrual, early proliferative, late proliferative, and secretory phases, respectively. At the same time, the intensity score was 96.735 (±26.24), 117.4 (±27.8), 145.37 (±30.0137), and 157.3 (±21.3) in these phases. Endometrial thickness also showed a moderate positive correlation with the intensity score (r=0.545, p=0.000).

**Discussion:**

These findings, which demonstrate a correlation between the intensity score and endometrial thickness, underscore the importance of this study in providing a basis for using ImageJ software to analyze transabdominal ultrasound.

**Conclusion:**

This pilot study generated preliminary reference values for endometrial thickness and intensity score using transabdominal ultrasound. It also demonstrated a correlation between these measurements, underscoring the potential utility of ImageJ analysis.

## 1 INTRODUCTION

The endometrium, the inner lining of the uterus, is dynamic and highly sensitive to changes in the levels of sex steroid hormones, including ovarian estradiol and progesterone [1]. During the menstrual cycle, the endometrium undergoes a sequence of hormonal changes. Initially, it is exposed to systemic estradiol, followed by a combination of estradiol and progesterone. If pregnancy does not occur, there is a subsequent withdrawal of progesterone. Due to this consecutive exposure, the endometrium changes both structure and function throughout the menstrual cycle [2]. The various stages of the monthly menstrual cycle are caused by the interplay between sex hormones and the responses of the endometrium [3].

Precise assessment of endometrial thickness is essential for the evaluation of different physiological and pathological states in the uterus, including uterine fibroids, polyps, and endometrial hyperplasia [4-6]. For this purpose, sonographic assessment is a widely used non-invasive method, as it is accessible, safe, and effective in providing detailed visualization of pelvic structures [7, 8].

In gynecology, transvaginal sonography (TVS) is the procedure of choice for evaluating the endometrium [9], but in Arab countries, transvaginal ultrasound cannot be used for singles. So, transabdominal sonography (TAS), which has a low resolution, is used as an alternative technique.

Different computer-assisted image analysis (CAIA) software has recently been developed to analyze different images [10]. Various studies confirmed the usefulness of this postprocessing software in assessing different conditions. One study approved the utility of this postprocessing software in assessing the physiological state of the endometrium [11]. ImageJ was found to be useful in differentiating benign from malignant ovarian masses [12]. A preliminary clinical investigation used a curvelet transform-based texture analysis of carotid B-mode ultrasound images in asymptomatic males with moderate to severe stenosis. The results indicated that the curvelet transform is a promising method for identifying novel markers of cardiovascular risk [13]. CAIA software, such as Texture Analysis and ImageJ (formerly known as NIH Image), can measure the image's grey level by calculating pixel intensity. Utilizing technologies, such as computer-assisted image analysis (CAIA) software for three-dimensional ultrasonography to interpret the findings of TAS, may enhance the accuracy of assessing uterine anatomy and endometrial conditions [3].

In this study, we examined the usefulness of ImageJ software in analyzing ultrasonographic images of the uterus to assess the physiologic states of the endometrium in normal menstrual cycles among single females in KSA. The specific objectives of this study were to quantify endometrial layering using ImageJ software, to evaluate differences in the mean values of endometrial layering, and to assess the correlation between endometrial thickness and the quantified layering.

## 2 MATERIALS AND METHODS

### 2.1 Study Design and Setting

This was a longitudinal cohort design assessing the endometrium for each participant in all four menstrual phases. The study sample comprised 20 volunteers recruited randomly at Princess Nourah University (PNU) ultrasound lab from November to April 2023. This was designed as a pilot normative cohort study to establish baseline values for endometrial thickness and echogenicity using TAS with ImageJ analysis. A sample of 20 healthy single females was considered adequate to demonstrate feasibility, refine the imaging and analysis protocol, and capture variability across the menstrual phases. This study included single females of childbearing age at PNU, aged 18 years and older, who had regular menstrual cycles lasting between 26 and 35 days. Those who have irregular menstrual cycles or any uterine problems, including structural or pathological conditions, such as congenital anomalies, fibroids, polyps, endometriosis, adenomyosis, congenital uterine anomalies, irregular menstrual cycles, polycystic ovarian syndrome, and other relevant gynecologic disorders, were excluded.

### 2.2 Ultrasound Scan and Data Collection

A Philips iU22 Ultrasound Machine performed pelvic scanning with a low-frequency probe according to the guidelines of the American Institute of Ultrasound in Medicine [14]. Each volunteer was scanned in four phases, yielding a total of 80 scans for analysis. This preliminary dataset provides reference values and supports the design of larger studies in similar populations. Scanning was performed by seven sonographers under the supervision of an experienced ultrasound specialist. All operators received standardized training and adhered to a unified scanning protocol. Although formal interobserver reliability metrics were not calculated, consistency was promoted through the use of predefined imaging parameters and regular supervision. The menstrual stage of each volunteer was determined based on the self-reported last menstrual period (LMP) and cycle length. Females with a gestation length between 26 and 35 days were included to minimize variability. In a typical 28-day cycle, the menstrual phase occurs on days 1–5, followed by the early proliferative phase on days 6–9, the late proliferative phase on days 10–14, and finally the secretory phase on days 15–28. For cycles of different lengths, the individual differences were calculated proportionally according to the following equation:

Adjusted day= Standard day in 28-day cycle * Volunteer’s cycle length/ 28

For example, if a participant had a cycle length of 33 days, the menstrual phase would occur on days 1–6, the early proliferative phase on days 7-11, the late proliferative phase on days 12-16, and finally the secretory phase on days 17-33.

Volunteers were asked to come with a full bladder, then pelvic scanning was performed longitudinally, and a sagittal section clearly showed the endometrium. Endometrial thickness was measured at the widest section in the longitudinal view. Endometrial layering has four numerical values: one, three, five, or six layers. Typically observed during menstruation, a single layer presents as a single hyperechoic line. The three-layer pattern, commonly seen in the early proliferative phase, consists of one hyperechoic line flanked by two hypoechoic lines. The five-layer pattern, characteristic of the late proliferative phase, includes one hyperechoic line, two hypoechoic lines, and two additional hyperechoic lines. A six-layer configuration, characterized by a thickened endometrium in which all layers demonstrated hyperechogenicity and no discernible sublayers were observed during the secretory phase. The procedure was repeated in all phases of the menstrual cycle, and exact measurements were taken.

### 2.3 Data Analysis

**Fig. (1):**
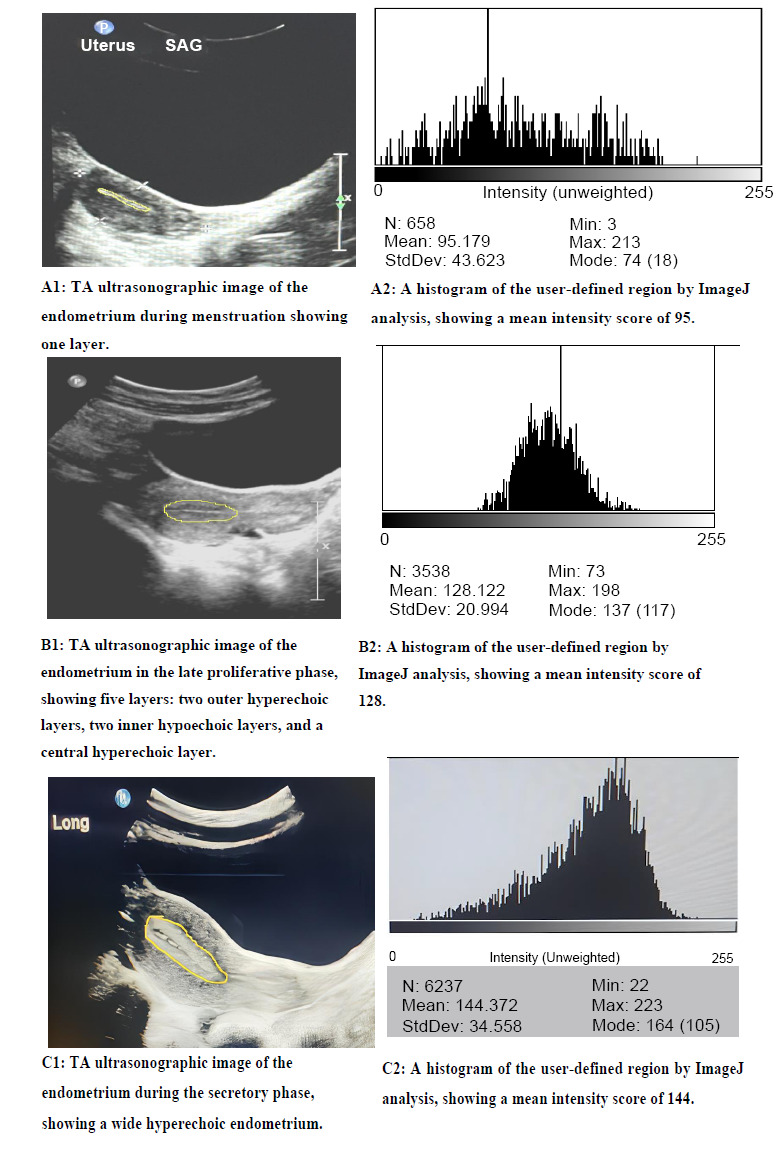
TAS and ImageJ assessment for different menstrual phases. (Images were with ImageJ (version 1.53k; Schneider *et al*., 2012)).

All images were saved and inserted into the ImageJ program (version 1.53k**;** Schneider *et al*., 2012) [15] to calculate image intensity at the region of the endometrium (Fig. **1**). All images were analyzed by a single operator. ImageJ analysis was performed using a standard procedure on all images. The region of interest (ROI) was manually delineated by drawing a cursor around the endometrial strip, carefully excluding background and non-target tissues. Mean gray values were then extracted to quantify pixel intensity.

Data were entered into Microsoft Excel and analyzed using SPSS V26, and the results were presented as mean ± standard deviation (SD). The difference between menstrual phases was tested using ANOVA, and the correlation between ultrasound measurements and intensity score was assessed using Pearson correlation.

## 3 RESULTS

### 3.1 Characteristics of Study Participants

The study included 20 females of reproductive age, with a mean age of 21 years (± 0.9). The average menstrual cycle length was 29.65 days (±2.18) (Table **1**).

### 3.2 Endometrial Measurements According to the Menstrual Phases

The endometrial thickness, layering, and intensity score varied significantly across different menstrual phases (Table **2**).

### 3.3 Endometrial Thickness

During the menstruation phase, the endometrium sheds, making the mean endometrial thickness 0.33cm (±0.137). In the early proliferative phase, it increased to 0.63 cm (±0.172). The late proliferative phase showed a further increase to 0.89 cm (±0.167). Finally, the secretory phase had the highest mean endometrial thickness at 1.06 cm (±0.19).

### 3.4 Endometrial Layers

The mean number of endometrial layers during menstruation was 1.25 (±1.118). Then it increased to 2.70 (±0.979) in the early proliferative phase. The late proliferative phase had a mean of 4.80 layers (±0.616), and the secretory phase showed the highest mean number of layers at 5.85 (±0.366).

### 3.5 Intensity Score

The mean intensity score during the menstruation phase was 96.735 (±26.24). In the early proliferative phase, it increased to 117.4 (±27.89). The late proliferative phase had a mean intensity score of 145.37 (±30), and the highest intensity score was observed in the secretory phase at 157.300 (±21.3).

### 3.6 Summary Statistics by Menstrual Phases

A further breakdown of intensity scores by menstrual phase is provided in Fig. (**2**).

- During the early proliferative phase, the mean intensity score was 117.405 (±27.88794), with a minimum of 62.5 and a maximum of 159.2.
- In the late proliferative phase, the mean intensity score increased to 145.37 (±30.01372), ranging from 63.5 to 198.6.
- The secretory phase had a mean intensity score of 157.3 (±21.30987), with values ranging from 108.4 to 196.3.
- During menstruation, the mean intensity score was 96.735 (±26.2434), with a minimum of 56 and a maximum of 137.6.

### 3.7 Correlation Analysis

Correlation analysis showed a significant correlation among endometrial measurements (Table **3**). There was a strong positive correlation between endometrial thickness and layering (r=0.788, p=0.000), and endometrial thickness also showed a moderate positive correlation with the intensity score (r=0.545, p=0.000). Similarly, there was a moderate positive correlation between endometrial layering and the intensity score (r=0.591, p=0.000).

## 4 DISCUSSION

ImageJ is capable of objective quantification. It fundamentally quantifies images by mathematically assessing pixel values. ImageJ can objectively measure parameters, such as size and area, and mean pixel intensity. Once measurement parameters are defined, the software applies them uniformly across all images, resulting in reproducible and unbiased numerical outputs [16, 17]. ImageJ analysis is efficient, reproducible, conserves time, and maintains uniformity across extensive datasets [18]. Whereas ultrasound interpretation relies heavily on subjective operator judgment, ImageJ can potentially reduce interobserver variability and human error.

This study examined a crucial issue, which is the gynecological sonographic evaluation of single females in Arab countries. In these countries, transvaginal ultrasound, the standard method, is not applicable. Therefore, we hypothesized that ImageJ software could be combined with TAS to enhance its diagnostic yield. Computer-assisted analysis, such as ImageJ, has been previously combined with transvaginal sonography, and it has been evident that this approach provides a more comprehensive analysis of the endometrium [19, 20]. This study included 20 volunteers; all recruited from a single center. As a pilot study, its primary aim was to assess the feasibility of ImageJ-based endometrial analysis rather than to generate fully generalizable results.

Regarding the endometrial thickness, it ranged from 0.33cm in the menstrual phase to 0.63 cm, 0.89 cm, and 1.06 cm in the early proliferative, late proliferative, and secretory phases, respectively. These measures correspond well with the references set by Sharma *et al*. [21].

Regarding the intensity score, the study revealed that image intensity varied according to the menstrual phase, with the secretory phase exhibiting the highest intensity and the menstrual phase showing the lowest intensity. These findings are in congruence with the results of a study by Chou *et al*., which support the monthly changes [11]. However, it is important to note that this study utilized TAS, whereas Chou *et al*. utilized transvaginal ultrasound; thus, there is a significant variation in their intensity score estimates.

These correlations align with the synchronized effect of cyclical hormonal changes on the endometrium. Estrogen promotes endometrial development, resulting in increased thickness, enhanced layering (the number of layers identifiable by ultrasound grows from one layer during the menstrual phase to a broad endometrium in the secretory phase), and an increase in echogenicity. During the luteal phase, progesterone further increases stromal density, vascularity, and glandular secretion, resulting in higher intensity scores. Therefore, the observed correlations reflect the parallel action of ovarian hormones on both structural features (like thickness and layering) and textural features (such as intensity) of the endometrium.

From a diagnostic perspective, integrating layering and intensity patterns with thickness may improve diagnostic accuracy in differentiating normal cyclical changes from pathological conditions, such as endometrial hyperplasia.

These findings, with the correlation between the intensity score and endometrial thickness, underscore the importance of this study in providing a ground for using ImageJ software to analyze TAS images. ImageJ software can help clinicians make more informed decisions regarding patient management, particularly in cases involving endometrial pathologies in single Arabian females. As the sensitivity and accuracy of the procedure improve, its clinical value increases as well. Comparing the findings with other gold standards, such as TVS, is strongly recommended. Additionally, since TAS has relatively low resolution, this may impact ImageJ calculations. Therefore, technical optimization, such as using the highest possible frequency, applying noise reduction, and enhancing contrast, must be followed.

## 5 LIMITATIONS

As this is a pilot study, several limitations should be acknowledged. The first notable limitation is the lack of an interobserver reliability assessment, as all ultrasound acquisitions were performed by seven operators. This restricts the generalizability of the findings. In real-world clinical practice, multiple sonographers and analysts are often involved, and subtle differences in probe positioning, image optimization, or region-of-interest selection could affect the measured parameters. Without measuring interobserver agreement, it is impossible to determine whether the observed values and correlations would stay consistent across different examiners. Future research should include blinded, independent assessments by multiple observers and report agreement statistics (e.g., intraclass correlation coefficients) to enhance the reproducibility and external validity of the results.

Second, the sample size was relatively small, and all participants were healthy volunteers recruited from a single university, with an age range concentrated around 21 years. This introduces selection bias and limits the generalizability of the findings to other age groups and populations with diverse socioeconomic backgrounds. Third, there was no comparison with a gold standard reference, such as TVS or histopathological findings, which restricts the ability to validate the measurement accuracy of the proposed method.

## CONCLUSION

In conclusion, this study provides vital information for gynecological assessment in general and for single females in Arabian countries in particular. The study indicated that the endometrial measurements using TAS were consistent with published reference values. Additionally, the study emphasizes the statistically significant correlation between intensity score and endometrial thickness. Future research should be conducted on a larger scale, involving a large number of participants at different centers, to compare the method with TVS as a gold standard and ensure further assessment of its reliability.

## Figures and Tables

**Fig. (2):**
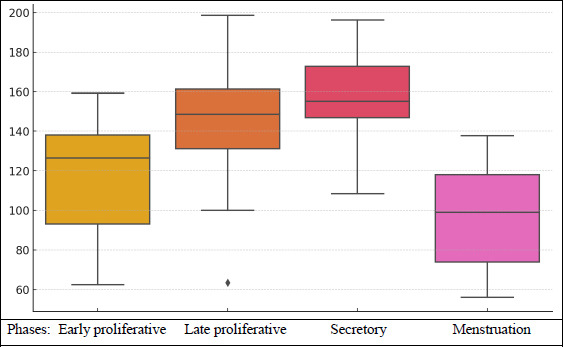
Intensity score according to the menstrual phases.

**Table 1:** Characteristics of study participants.

Measure	Mean± SD
Age (years)	21± 0.9
Menstrual length (days)	29.65± 2.188

**Table 2:** Endometrial measurements according to the menstrual phases.

**Parameter**	**Menstruation** **(Mean ± SD)**	**Early Proliferative** **(Mean ± SD)**	**Late Proliferative** **(Mean ± SD)**	**Secretory** **(Mean ± SD)**	**F**	***P*-value**
Endometrial Thickness (cm)	0.33±0.14	0.63±0.17	0.90±0.17	1.06±0.19	72.892	0.000
Endometrial Layers (n)	1.25 ± 1.12	2.70 ± 0.98	4.80 ± 0.62	5.85 ± 0.37	125.687	0.000
Intensity Score (a.u)	96.74 ± 26.24	117.41 ± 27.89	145.37 ± 30.01	157.30 ± 21.31	21.211	0.000

**Table 3:** Correlation among endometrial measurements.

**-**		**Thickness**	**Layering**	**I score**
Thickness	Pearson Correlation	1	0.788**	0.545**
	Sig. (2-tailed)		0.000	0.000
Layering	Pearson Correlation	0.788**	1	0.591**
	Sig. (2-tailed)	0.000		0.000
Intensity score	Pearson Correlation	0.545**	0.591**	1
	Sig. (2-tailed)	0.000	0.000	

Note: **. Correlation is significant at the 0.01 level (2-tailed).

## Data Availability

The data and supportive information are available within the article.

## List of Abbreviations

CAIA: Computer-Assisted Image Analysis
LMP: Last Menstrual Period
PNU: Princess Nourah University
ROI: Region of Interest
SD: Standard Deviation
TAS: Transabdominal Sonography
TVS: Transvaginal Sonography

## STANDARDS OF REPORTING

STROBE guidelines were followed.
